# Novel Cannabinoid Receptor 2 (CB2) Low Lipophilicity Agonists Produce Distinct cAMP and Arrestin Signalling Kinetics without Bias

**DOI:** 10.3390/ijms24076406

**Published:** 2023-03-29

**Authors:** Raahul Sharma, Sameek Singh, Zak M. Whiting, Maximilian Molitor, Andrea J. Vernall, Natasha L. Grimsey

**Affiliations:** 1 Department of Pharmacology and Clinical Pharmacology, School of Medical Sciences, Faculty of Medical and Health Sciences, University of Auckland, Auckland 1023, New Zealand; raahul.sharma@auckland.ac.nz (R.S.);; 2 Centre for Brain Research, Faculty of Medical and Health Sciences, University of Auckland, Auckland 1023, New Zealand; 3 Department of Chemistry, University of Otago, Dunedin 9016, New Zealandmolitor@pharmchem.uni-frankfurt.de (M.M.); andrea.vernall@otago.ac.nz (A.J.V.); 4 Chemical Biology and Therapeutics Science, Broad Institute of MIT and Harvard, Cambridge, MA 02142, USA; 5 Institute of Pharmaceutical Chemistry, Goethe University, 60438 Frankfurt, Germany; 6 Maurice Wilkins Centre for Molecular Biodiscovery, Auckland 1142, New Zealand

**Keywords:** receptor, cannabinoid, CB2, cannabinoid receptor agonists, signal transduction, cyclic AMP, beta-arrestin 2, kinetics, drug development, chemistry, pharmaceutical

## Abstract

Cannabinoid Receptor 2 (CB2) is a promising target for treating inflammatory diseases. We designed derivatives of 3-carbamoyl-2-pyridone and 1,8-naphthyridin-2(1H)-one-3-carboxamide CB2-selective agonists with reduced lipophilicity. The new compounds were measured for their affinity (radioligand binding) and ability to elicit cyclic adenosine monophosphate (cAMP) signalling and β-arrestin-2 translocation with temporal resolution (BRET-based biosensors). For the 3-carbamoyl-2-pyridone derivatives, we found that modifying the previously reported compound UOSS77 (also known as S-777469) by appending a PEG2-alcohol via a 3-carbomylcyclohexyl carboxamide (UOSS75) lowered lipophilicity, and preserved binding affinity and signalling profile. The 1,8-naphthyridin-2(1H)-one-3-carboxamide UOMM18, containing a *cis* configuration at the 3-carboxamide cyclohexyl and with an alcohol on the 4-position of the cyclohexyl, had lower lipophilicity but similar CB2 affinity and biological activity to previously reported compounds of this class. Relative to CP55,940, the new compounds acted as partial agonists and did not exhibit signalling bias. Interestingly, while all compounds shared similar temporal trajectories for maximal efficacy, differing temporal trajectories for potency were observed. Consequently, when applied at sub-maximal concentrations, CP55,940 tended to elicit sustained (cAMP) or increasing (arrestin) responses, whereas responses to the new compounds tended to be transient (cAMP) or sustained (arrestin). In future studies, the compounds characterised here may be useful in elucidating the consequences of differential temporal signalling profiles on CB2-mediated physiological responses.

## 1. Introduction

Cannabinoid Receptor 2 (CB2), a G protein-coupled receptor (GPCR), performs critical roles in regulating inflammation, bone mass, and nociception, without mind-altering properties that are associated with activation of Cannabinoid Receptor 1 (CB1). As such, therapeutic modulation of CB2 is considered a promising treatment strategy in various contexts. This has been explored with success in multiple preclinical in vivo studies [1].

However, designing CB2 ligands that can translate into the clinic is challenging and, in the few human clinical trials to date, the efficacy of CB2-targeted compounds has been marginal [2]. With similar orthosteric ligand-binding pockets between CB1 and CB2, achieving highly selective CB2 ligands poses difficulty [3]. From a physiochemical standpoint, most established cannabinoids are very lipophilic. This implies likelihood of permeability through the blood–brain barrier into the central nervous system, and subsequent potential risk of engagement with CB1 and psychoactivity [4]. Concurrently, highly lipophilic compounds are associated with greater risk of toxicity, and are poor candidates for oral administration due to limited intestinal absorption. Moreover, it is established that agonists acting via CB2 can produce signalling bias (biased agonism / functional selectivity), and that different subcellular populations of CB2 may have distinct coupling to signalling mediators [2]. Both of these aspects have potential to influence the physiological impact of CB2-activating compounds, and therefore, are important to consider in drug design and characterisation. To address these challenges, a variety of cannabinoid receptor-targeting scaffolds have been optimised for greater polarity and CB2 selectivity, though they have undergone relatively little characterisation as yet, e.g., [5,6,7].

Here, we contribute to efforts in the development and characterisation of CB2-selective ligands with considerably different physicochemical properties to established cannabinoids that can act as useful chemical tools and that may be favourable for a peripherally acting drug. The novel compounds reported herein were designed to be more polar derivatives of two previously reported CB2-selective agonist scaffolds, 3-carbamoyl-2-pyridone and 1,8-naphthyridin-2(1H)-one-3-carboxamide (Table 1). UOSS77 (first reported as S-777469, [6,8]) is a 3-carbamoyl-2-pyridone analogue with reduced lipophilicity (cLogP, cLogD_7.4_) in comparison with established cannabinoids (for example, CP55,940; Table 1). UOSS77 was previously reported as a CB2-selective agonist [6]. UOSS77 is orally bioavailable and has shown promise as an anti-pruritic agent in rodents, though was informally reported to be ineffective in Phase 2 clinical trial for atopic dermatitis [6,9,10,11]. Other relatively polar 3-carbamoyl-2-pyridone derivatives have also been reported as CB2 agonists, but with less selectively for CB2 over CB1 compared to UOSS77, for example, S-444823 [12]. Subsequently, polar CB1/CB2 dual agonists were produced by addition of polar groups, such as alcohol and sulphonamide moieties from the 3-position of the pyridone core (for numbering refer to UOSS77, Table 1) [13]. Inspired by this approach and based on the more CB2 selective scaffold of UOSS77, we report two novel more polar derivatives (decreased cLogP) of UOSS77 that contain a polyethylene glycol 2 alcohol (PEG2-alcohol) (UOSS75) or polyethylene glycol 3 alcohol (PEG3-alcohol) (UOSS76) moiety, introduced via an amide bond from the 3-carbomylcyclohexyl carboxylic acid of UOSS77.

The 1,8-naphthyridin-2(1H)-on-3-carboxamide scaffold was first reported as a cannabinoid receptor agonist, and it was demonstrated that the *cis* configuration of a methylcyclohexyl extending from the 3-carboxamide had particular promise (for numbering refer to UOMM18, Table 1) [5]. A further comprehensive study of ‘R’ groups around the 1,8-naphthyridin-2(1H)-on-3-carboxamide scaffold showed that substitution from the ‘pyridine-like’ ring could switch from agonist to antagonist/inverse agonist action and that various N1-alkyl substituents, including morpholinoethyl, were well tolerated for CB2 activity [14]. Compounds with this scaffold have exhibited immunomodulatory properties in in vitro models (for example ‘VL15′ [15]), and have been developed into fluorescent CB2 ligands (compound 32 in [16]). Based on structure-activity relationships from previous reports [5,14], and with the driver to make CB2 selective ligands with reduced lipophilicity, herein, we designed and synthesised UOMM18 (Table 1), with the *cis* configuration at the 3-carboxamide cyclohexyl and with an alcohol on the 4-position of the cyclohexyl to lower lipophilicity.

Compounds UOSS77, UOSS75, UOSS76, and UOMM18 were synthesised and characterised for their CB2 and CB1 binding affinities, potency, and efficacy for inducing cAMP signalling and β-arrestin-2 recruitment, and potential for producing biased agonism. We studied signalling under both a “traditional” paradigm, via concentration response curves for the overall response to a 20 min stimulation, and over time during the 20 min stimulation.

## 2. Results

### 2.1. Compound Properties and Binding Affinities

Two novel derivatives of UOSS77, that contain additional polar functional groups added via the 3-carbomylcyclohexyl carboxylic acid of UOSS77, were synthesised; a PEG2-alcohol (UOSS75) or PEG3-alcohol (UOSS76) (Table 1, refer to Supplementary Information for synthesis and compound characterisation). The physicochemical properties of UOSS75 and UOSS76 were calculated, and have a reduced lipophilicity (lower cLogP and higher TPSA) compared to the parent compound UOSS77. However, at physiological pH 7.4, UOSS77 has a lower cLogD_7.4_ than UOSS75 and UOSS76 by virtue of the predominantly ionised carboxylic acid functional group that UOSS75 and UOSS76 lack.

UOMM18 (Table 1), a novel, less lipophilic 1,8-naphthyridin-2(1H)-on-3-carboxamide, was also synthesised (refer to Supplementary Information for synthesis and compound characterisation), and the physicochemical properties were calculated. Compared to established cannabinoids, UOMM18 has lower lipophilicity (decreased cLogP, increased TPSA), including at physiological pH 7.4 (cLogD_7.4_) (Table 1).

**Figure 1:**
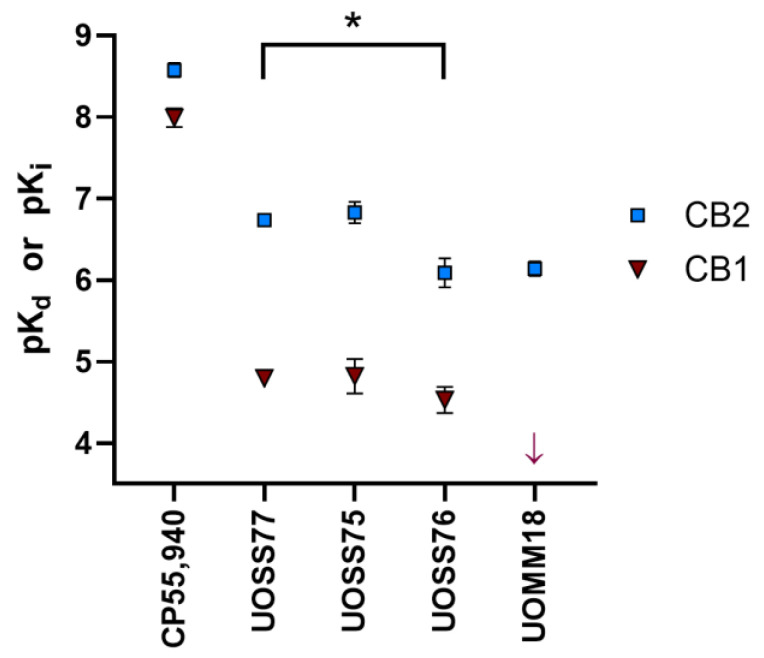
Binding affinities of compounds of interest at human CB2 or CB1 determined from radioligand competition binding assays (pK_d_ for CP55,940, pK_i_ for other compounds). Data are mean ± SEM from three to four independent experiments, except for UOSS75 at CB1 (*n* = 2). CB1 pK_i_ values for all compounds, other than CP55,940, are estimates (see main text and Table 1). The arrow for UOMM18 in the CB1 set indicates no detectable binding to the lowest possible affinity constant measurable in our assay (pK_i_ < 4). CB2 affinity was statistically compared between UOSS77 and its novel derivatives; * represents *p* < 0.05.

We measured the binding affinities of the compounds of interest to human CB2 and CB1 via radioligand binding assay. All 3-carbamoyl-2-pyridones (UOSS77, UOSS75, and UOSS76) fully displaced [^3^H]-CP55,940 from CB2, but had moderate affinities that were lower than CP55,940 (Table 1, Figure 1). Compared to UOSS77, UOSS75 had a similar affinity for CB2, whereas UOSS76 had a significantly reduced CB2 pK_i_ (by 0.6 log units vs. UOSS77; *p* = 0.015). At CB1, only partial displacement of [^3^H]-CP55,940 was detected even at the highest applied concentration of the test compounds (31.6 µM). Therefore, we utilised the point of full displacement by CP55,940 to assist in fitting displacement curves, and to consider the CB1 affinity parameters for the UOSS series compounds to be estimates. Affinities of UOSS77, UOSS75, and UOSS76 at CB1 all appeared to be similar, and the low affinity at CB1 implied that all the 3-carbamoyl-2-pyridones tested were CB2-selective.

UOMM18 had moderate affinity for CB2, but this was considerably lower than the pK_d_ of CP55,940 by ~2.5 log units (*p* < 0.0001). UOMM18 did not detectably displace [^3^H]-CP55,940 at CB1 and, based on our experimental design, we estimated that if UOMM18 has any affinity for CB1 the affinity constant must be greater than 100 µM. Lack of measurable affinity at CB1 resulted in UOMM18 having the greatest CB2 selectivity of the compounds tested (Table 1, Figure 1).

### 2.2. cAMP Signalling

A real-time bioluminescence resonance energy transfer (BRET)-based CAMYEL cAMP biosensor was used to measure the effect of CP55,940 and all test compounds on forskolin-stimulated cAMP levels. As CB2 is generally observed to couple to Gα_i_, we expected CB2 agonists to reduce cAMP relative to the forskolin-induced level.

**Figure 2:**
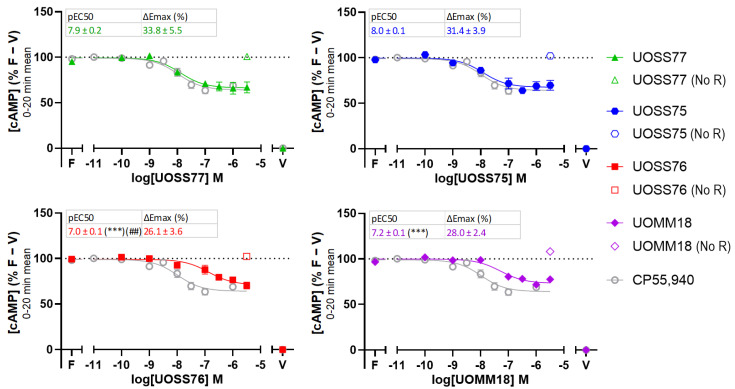
cAMP levels in response to varying concentrations of compounds of interest co-stimulated with 5 μM forskolin in cells transfected with human CB2 or control plasmid (“No R”). Biosensor measurements over a 20 min drug treatment were averaged, then normalised to vehicle-treated without forskolin (V; 0%) and vehicle-treated with forskolin (F; 100%) conditions. pEC_50_ and E_max_ are displayed in boxes for test compounds. E_max_ is expressed as absolute magnitude percentage decrease from 100% (ΔE_max_), such that larger ΔE_max_ represents greater efficacy. CP55,940 has pEC_50_ 8.0 ± 0.1 and ΔE_max_ 35.1% ± 3.5%. Plotted and parameter data are mean ± SEM from three to four independent experiments. Asterisks and hashes represent a significant difference from the parameters for CP55,940 and UOSS77, respectively. ## and *** represent *p* < 0.01 and < 0.001, respectively.

We first measured the cAMP response to varying concentrations of ligand by analysing the mean of measurements taken during a 20 min stimulation (Figure 2). All 3-carbamoyl-2-pyridones and UOMM18 produced typical CB2 agonist concentration responses, indicated by a sigmoidal decrease in cAMP levels with greater drug concentrations. The efficacies of the test compounds were all similar to each other and to CP55,940 (no significant differences), suggesting they exhibit full agonism in the cAMP signalling pathway. UOSS77 and UOSS75 produced similar potency responses as CP55,940, whereas UOSS76 and UOMM18 had lower potencies, indicated by a decrease in pEC_50_ in comparison with CP55,940 of ~1.0 and ~0.8 log units, respectively (*p* < 0.001, 0.001). All test compounds were also evaluated for non-CB2-mediated effects by application to cells that were not transfected with CB2 (“No R” controls). cAMP levels did not change when the highest concentration (3.16 µM) of each compound was tested, suggesting the cAMP responses in cells expressing CB2 were likely mediated via CB2.

**Figure 3:**
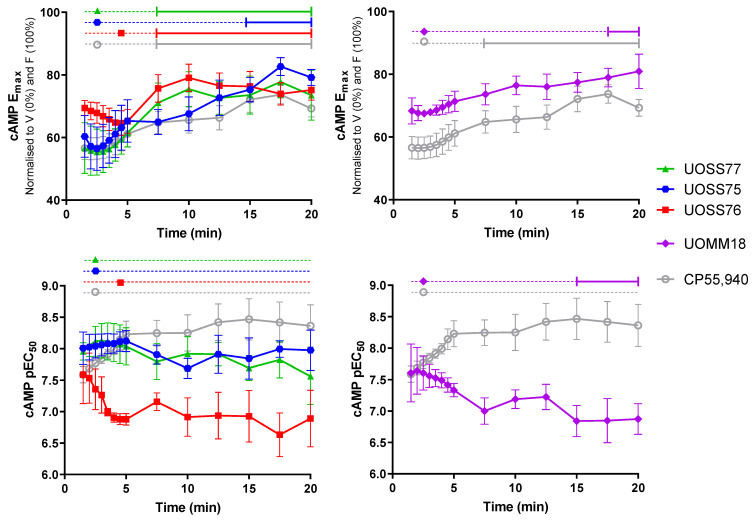
cAMP signalling efficacy (E_max_; top panels) and potency (pEC_50_; bottom panels) at different time-points during 20 min stimulation with compounds of interest and 5 μM forskolin. Parameters were derived from concentration response curves generated at corresponding time-points with data normalised to vehicle-treated without forskolin (V; 0%) and vehicle-treated with forskolin (F; 100%), as in Figure 2 and Figure S1, such that smaller cAMP measurements indicate greater efficacy (inhibition of cAMP synthesis). Data are mean ± SEM from three to four independent experiments. Horizontal lines across the top of plots indicate results of within-drug statistical comparisons to the reference time-point (maximal efficacy), which is indicated by the dataset symbol. Solid capped lines indicate significant difference from the reference time-point (*p* < 0.05), whereas dotted lines indicate no significant difference from the reference time-point.

An advantage of utilising real-time signalling biosensors is that these enable analysis of the kinetic nature of functional responses. Having established that the test compounds acted as agonists when the response was aggregated over time, we undertook kinetic analysis of the cAMP responses by generating concentration response curves at multiple points during the 20 min stimulation period, and deriving parameters of pEC_50_ and E_max_ for each (Figure 3, Figure S1). Data were normalised to forskolin-stimulation (100%) and vehicle-treated without forskolin (0%). Therefore, smaller E_max_ values represent more efficacious responses, as these indicate greater reduction in cAMP relative to forskolin-stimulation.

CP55,940 produced a peak response within 2.5 min of stimulation that then gradually diminished back toward forskolin alone, becoming significantly lesser than the maximal response from 7.5 min (*p* = 0.033). A partial response was present throughout the remainder of the measured time-course, that is, the cAMP level did not return to that induced by forskolin alone. UOSS77, UOSS75, and UOMM18 exhibited similar efficacy profiles to CP55,940, reaching maximal efficacy 2.5 min with responses, then reduced gradually, reaching significantly lesser E_max_ than at 2.5 min starting from 7.5 min, 15 min, and 17.5 min, respectively (*p* = 0.018, 0.020, 0.026). UOSS76 took longer to produce a peak response at around 4.5 min, which then decreased significantly to a partial response from 7.5 min (*p* = 0.001).

The approximate peak response time for each compound was then used as the reference point for comparing potencies over time (Figure 3). The potency of response to CP55,940 appeared to increase during the acute 5 min period of maximal efficacy (by ~0.5 log units), though no significant differences from the potency at 2.5 min were detected. The CP55,940 response potency then remained consistent throughout the rest of the 20 min time-course. Within the 3-carbamoyl-2-pyridone set, UOSS77 and UOSS75 response potencies were similar, both to each other and over time, remaining stable throughout the entire 20 min stimulation. Conversely, the potency of response to UOSS76 decreased during the early phase of stimulation (by ~0.7 log units), though no time-points were significantly different from the potency at 4.5 min. Despite the lack of statistically distinguishable changes in potency over time within both the UOSS76 and CP55,940 datasets, the divergent trends were statistically evident when comparing between these compounds. Whereas the potencies of UOSS76 and CP55,940 were equivalent at early time-points, the pEC_50_ values became significantly different from 4 min (*p* = 0.038). UOMM18′s potency also diminished over time, becoming significantly different from the potency at 2.5 min from the 15 min time-point (*p* = 0.014). Initial response potencies were again equivalent between UOMM18 and CP55,940, but diverged over time, becoming significantly different from 7.5 min (*p* = <0.001 to 0.022, except 10 min no significant difference, *p* = 0.063).

The similarity of E_max_ temporal trajectories between ligands denotes that when applied at sufficiently high ligand concentrations (maximal at all time-points), the kinetic nature of responses will also be similar. However, divergences in EC_50_ trends over time imply that sub-maximal concentrations will elicit responses with differing kinetics between ligands. For example, consider the efficacy over time of a single concentration that is near the EC_50_ at an early time-point. The increasing potency of the CP55,940 response implies that this concentration becomes relatively more effective over time, trending toward E_max_. In contrast, decreasing potencies for UOSS76 and UOMM18 over time imply that progressively greater ligand concentrations are required to elicit maximal responses. Therefore, a ligand concentration near the initial EC_50_ will tend toward producing a reduced response relative to the E_max_ over time. UOSS77 and UOSS75 sit between these two extremes, having relatively stable potencies over time. As such, response kinetics tend to mirror the E_max_ trend at all effective ligand concentrations. These profiles are illustrated by comparative concentration response curves at different time-points, and response time-courses for 2.5 min EC_75_ concentrations versus E_max_ in Figure S1.

### 2.3. β-Arrestin-2 Recruitment

**Figure 4:**
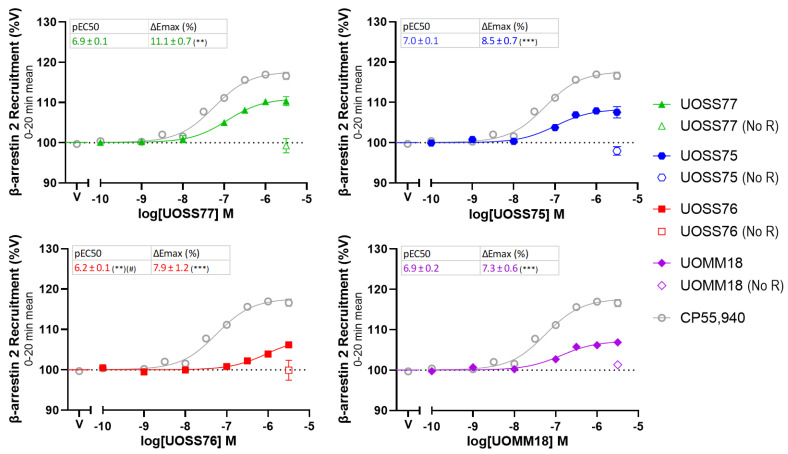
β-arrestin-2 plasma membrane translocation induced by varying concentrations of compounds of interest, in cells transfected with human CB2 or control plasmid (“No R”). Biosensor measurements over a 20 min drug treatment were averaged, then normalised to vehicle-treated (V; 100%). pEC_50_ and ΔE_max_ (% increase above V) are displayed in boxes for test compounds. CP55,940 has pEC_50_ 7.2 ± 0.1 and ∆E_max_ 17.6% ± 0.8%. Plotted and parameter data are mean ± SEM from three independent experiments. Asterisks and hashes represent a significant difference to the parameters of CP55,940 and UOSS77, respectively. #, ** and *** represent *p* < 0.05, 0.01 and 0.001, respectively.

Another real-time BRET-based biosensor assay was conducted to measure recruitment of β-arrestin-2 to the plasma membrane in response to the compounds of interest relative to vehicle-treated cells over a 20 min duration (Figure 4). In cells expressing CB2, all compounds showed sigmoidal increases in β-arrestin-2 recruitment from baseline with greater drug concentrations, typical of CB2 agonism. However, the efficacy of all test compounds was lower than CP55,940, suggesting they exhibit partial agonism for β-arrestin-2 translocation. A similar efficacy was shared between the 3-carbamoyl-2-pyridones. Although UOSS77 trended toward having greater efficacy, this did not reach statistical significance. All test compounds shared a similar potency to CP55,940, except for UOSS76, which induced a lower potency response, indicated by a smaller pEC_50_ (by ~1.0 log units; *p* = 0.003). Within the 3-carbamoyl-2-pyridone set, only UOSS76 had a weaker potency than UOSS77 (by ~0.7 log units; *p* = 0.017). Increases in β-arrestin-2 recruitment by test compounds were likely mediated via CB2 since no changes in the BRET ratio were detected when their highest concentration (3.16 µM) was applied to cells without transfected CB2 (“No R” controls).

**Figure 5:**
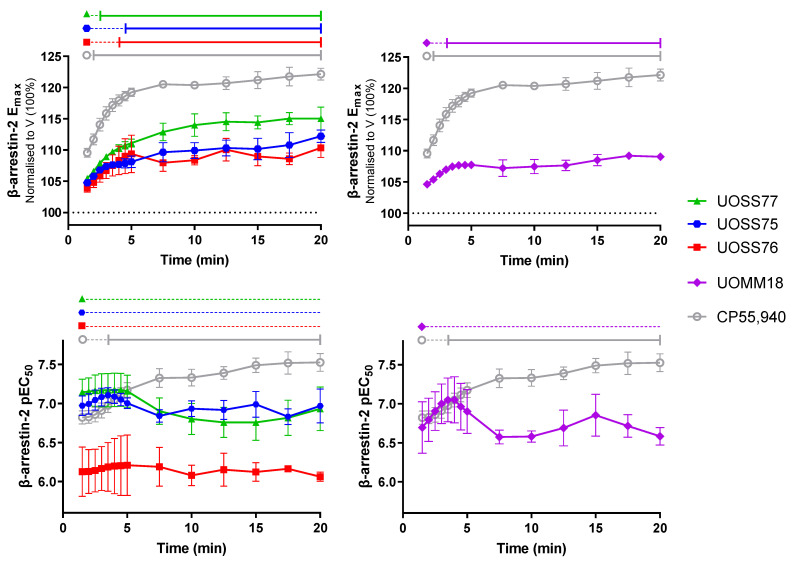
β-arrestin-2 plasma membrane translocation efficacy (E_max_; top panels) and potency (pEC_50_; bottom panels) at different time-points during a 20 min stimulation with compounds of interest. Parameters were derived from concentration response curves generated at corresponding time-points with data normalised to vehicle-treated (as in Figure 4 and Figure S2), then 100% subtracted (V; 100%), implying that larger E_max_ values indicate proportionally greater efficacy than the vehicle measurement. Data are mean ± SEM from three independent experiments. Horizontal lines across the top of plots indicate results of within-drug statistical comparisons to the reference time-point (first robust concentration response curve), which is indicated by the dataset symbol. Solid capped lines indicate significant difference from the reference time-point (*p* < 0.05), whereas dotted lines indicate no significant difference from the reference time-point.

Similarly as for cAMP signalling, kinetic analysis of β-arrestin-2 recruitment signalling was also conducted. Figure 5 shows E_max_ and pEC_50_ during a 20 min stimulation for CP55,940 and the test compounds. Changes in E_max_ were evaluated based on comparison to the responses at 1.5 min, as all compounds had sufficient efficacy at this first-measured point to produce robust concentration response curves and subsequently changed gradually without an obvious “peak” time-point. The efficacy of CP55,940 increased rapidly after the initial measurement and reached a plateau after approximately 5 min of stimulation. The test compounds exhibited similar temporal trajectories, but efficacies were partial in comparison with CP55,940. The relatively small response magnitudes for UOSS75, UOSS76, and UOMM18 correlated with these taking slightly longer to produce an increase in efficacy relative to that measured at 1.5 min. Efficacy for all ligands was fairly stable from around 5 min of stimulation through to the end of the 20 min measurement period.

Potencies over time were also compared to the 1.5 min time-point within each compound (Figure 5). The potency of response to CP55,940 increased during the stimulation, becoming significantly different from 3.5 min (*p* = 0.031), and overall increasing by ~0.7 log units during the stimulation. Conversely, test compounds maintained relatively stable potencies throughout the entire time-course (no significant differences from pEC_50_ at 1.5 min). CP55,940 and UOMM18 had equivalent potencies at early time-points, but diverged after the initial response, with the potency of CP55,940 stabilising, but UOMM18 decreasing, becoming significantly different from each other from 7.5 min (*p* = 0.003 to 0.049).

Interplay between efficacy and potency in the arrestin assay was reminiscent of findings for cAMP signalling. Although arrestin recruitment E_max_ kinetic trajectories were similar between ligands, differing potency trends over time imply that sub-maximal concentrations will tend to produce differing arrestin recruitment kinetics. CP55,940 potency tends to increase, and therefore, the response of a sub-maximal concentration will track toward E_max_ over time. In contrast, UOSS77, UOSS75, and UOMM18 potencies tend to decrease over time, so a sub-maximal concentration will produce progressively reduced response relative to E_max_. These profiles are illustrated by comparative concentration response curves at different time-points, and response time-courses for 1.5 min EC_75_ concentrations versus E_max_ in Figure S2. The contrasting profiles are most clearly demonstrated by comparing UOSS77 and CP55,940. Due to the weak partial agonism exhibited by UOSS75, UOSS76, and UOMM18, the response windows and differentials between EC_75_ response and E_max_ are small in both absolute magnitude and relative to assay variability.

### 2.4. Bias Analysis

**Figure 6:**
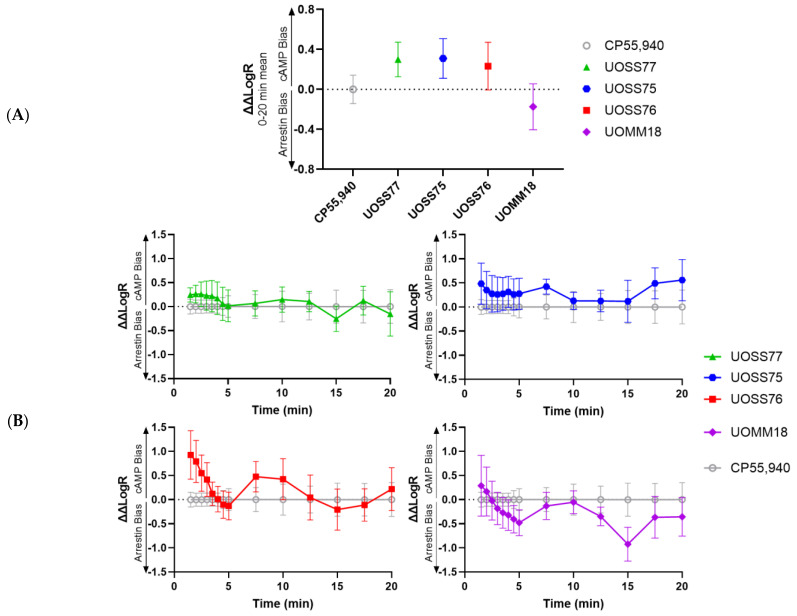
Signalling bias relative to CP55,940. Bias quantified as ΔΔLogR for cAMP signalling (positive; “cAMP Bias”) versus β-arrestin-2 translocation (negative; “Arrestin Bias”). (**A**) Bias for mean responses over a 20 min stimulation. (**B**) Bias at different time points throughout a 20 min stimulation. Data represents mean ± SEM from three to four independent experiments for each pathway.

Bias analysis utilising the Black and Leff operational model was conducted to determine whether any of the test compounds had greater preference for signalling via cAMP or β-arrestin-2 in comparison with the signalling profile produced by CP55,940 [7,17,18]. As for the functional assays, bias was analysed for the mean responses over a 20 min stimulation (Figure 6A), and for kinetic data in order to reveal the bias profile over time (Figure 6B). Following measurement of the transduction coefficient (LogR) for all independent concentration response curves in each signalling pathway, differences from the LogR for CP55,940 in the same pathway were determined (ΔLogR). The difference in ΔLogR between pathways for each drug (ΔΔLogR) then provided a quantitative measurement of the bias of a drug toward a particular pathway, relative to CP55,940′s signalling profile (ΔΔLogR = 0). None of the test compounds exhibited significant bias toward either pathway relative to CP55,940 in either the 20 min average analysis or at individual time-points (Figure 6A). In the kinetic analysis, statistical comparison to the first measurement at 1.5 min indicated that bias relative to CP55,940 remained relatively stable over time for all test compounds, though a trend of transition from cAMP bias toward arrestin bias was qualitatively observed for UOSS76 and UOMM18 (Figure 6B).

## 3. Discussion

We have characterised the molecular pharmacology of four CB2 ligands that are more polar in comparison with traditional cannabinoids; three 3-carbamoyl-2-pyridones and a 1,8-naphthyridin-2(1H)-one-3-carboxamide.

UOSS77, the 3-carbamoyl-2-pyridone set parent compound, had moderate CB2 affinity, which we measured to be somewhat lower than reported previously (S-777469 from [6], CB2 pK_i_ 7.4, error not shown). UOSS75 had equivalent affinity to UOSS77 indicating the PEG2-alcohol was tolerated, whereas PEG3-alcohol UOSS76 had somewhat reduced affinity. Taken together, this shows, as previously reported [6], that substitution from the 3-position of the pyridone core is tolerated. A speculative reason for the decrease in affinity going from PEG2-based UOSS75 to PEG3-based UOSS76 could be that larger molecular size and/or longer PEG linker perturb ligand entry; however, verification of this hypothesis would require complex ligand and receptor molecular dynamics to investigate. All 3-carbamoyl-2-pyridones behaved as full agonists in comparison with CP55,940 in the cAMP assay. Our result for UOSS77 was consistent with a previous report (from [6], agonism with pEC_50_ 7.6, error not shown). In alignment with affinity trends, PEG2-alcohol UOSS75 produced a similar cAMP response profile as UOSS77, whereas PEG3-alcohol UOSS76 produced overall lower potency cAMP signalling. To our knowledge, β-arrestin-2 recruitment had not previously been studied for 3-carbamoyl-2-pyridone CB2 agonists. These compounds all acted as partial agonists in comparison with CP55,940. In both signalling pathways, observation of kinetic signalling profiles revealed that temporal trajectories for maximal achievable efficacy were similar between ligands (including CP55,940), with the exception that UOSS76 exhibited slower acute cAMP signalling onset. However, divergence in temporal EC_50_ trajectories implied that application of sub-maximal ligand concentrations would produce ligand-specific temporal response patterns. Whereas CP55,940 tended to have sustained (cAMP) or increasing (arrestin) responses over time at sub-maximal concentrations, UOSS77, UOSS75, and UOSS76 exhibited reducing (cAMP) or sustained (arrestin) responses over time. Potential mechanisms and consequences of these temporal profiles are revisited below.

1,8-naphthyridin-2(1H)-one-3-carboxamide UOMM18 demonstrated similar general properties to previously reported structurally similar compounds, with lack of CB1 affinity and similar potency CB2 agonism for inhibiting cAMP synthesis [5,14]. The closest previously reported structural analogue “A1” in [14] (cLogP 1.49, cLogD_7.4_ 1.47), containing *cis*/*trans* 4-methyl instead of *trans*-4-hydoxy of UOMM18 (refer to Table 1 for numbering), was a partial agonist for cAMP inhibition, but full agonist for β-arrestin-2 recruitment relative to WIN55,212-2 [5,14]. We observed a trend toward UOMM18 acting as a partial agonist for cAMP inhibition, which was more evident in our detailed kinetic analysis of cAMP signalling. UOMM18 acted as a partial agonist for β-arrestin-2 recruitment relative to CP55,940. However, in comparing A1 from [14] and UOMM18, it is important to note that WIN55,212-2 is itself a weak partial agonist in comparison with CP55,940 for inducing β-arrestin-2 recruitment to CB2, which likely explains this apparent discrepancy [19]. Interestingly, in both the cAMP and arrestin assays, our detailed kinetic analysis revealed a similar temporal profile for UOMM18 to that described for the UOSS77, UOSS75, and UOSS76 above. Although E_max_ trajectories over time were similar between UOMM18 and CP55,940, diverging potencies implied that application of sub-maximal ligand concentrations produced responses with different temporal profiles. Due to the UOMM18 potency reducing over time, responses to sub-maximal concentrations tended to progressively degrade. In comparison, CP55,940 responses tended to be sustained. These findings are revisited below.

Given that the test compounds acted as full agonists in the cAMP pathway versus partial agonists in the β-arrestin-2 pathway (relative to CP55,940), we hypothesised that these may have bias toward cAMP signalling. Furthermore, our observation of differing kinetic trajectories between ligands led us to wonder whether bias may manifest differently over time as has been demonstrated for other GPCRs, e.g., [20]. However, no bias was statistically evident, whether analysed from averaged responses over a 20 min stimulation, or at individual time-points. For UOSS77 and UOSS75, this finding seems qualitatively consistent with the trends observed for the individual signalling assays in that overall EC_50_ trajectories were largely similar between cAMP and arrestin, the most obvious difference being the observed partial efficacy in the β-arrestin-2 assay. Lack of detection of bias may therefore imply that UOSS77 and UOSS75 are fundamentally partial agonists in both pathways we measured, but that they appeared to be full agonists in the cAMP assay due to receptor reserve [21,22,23]. Temporal bias analysis for both UOSS76 and UOMM18 qualitatively indicated a subtle trend for shifting bias over time from cAMP signalling (or lack of bias) toward β-arrestin-2 translocation. However, no statistical differences from CP55,940 were detected, and any underlying effect appeared to be small in magnitude. It is important to note that, although we did not detect bias between cAMP signalling and β-arrestin-2 translocation in our study, we cannot rule out that bias for signalling via other effectors could be present and/or that subtle biases in receptor-proximal signalling responses or arrestin conformation could manifest as bias in downstream responses [24].

Our results indicate that UOSS77, UOSS75, UOSS76, and UOMM18 are able to activate CB2 as high potency partial agonists with unique signalling kinetics in comparison with “traditional” CB2 full agonist CP55,940, and these kinetic differences were reflected in both the cAMP and β-arrestin-2 pathways (hence, lack of bias). This seemingly implies that ligands UOSS77, UOSS75, UOSS76, and UOMM18 ultimately stabilise similar complement(s) of receptor active conformation(s) as each other but must exhibit differing temporal profiles for stabilisation [25].

The most straightforward potential explanation for this phenomenon relates to differing binding kinetics between ligands. Slower dissociation rates (but not equilibrium binding affinity nor association rates) have been found to correlate with prolonged signalling responses and increasing potency over time at other GPCRs [20,26]. Interestingly, a correlation between increased lipophilicity and reduced dissociation rate has been reported for CB2 ligands, though this relationship was not dependent on ‘overall’ ligand lipophilicity, but the lipophilicity of varying ‘R’ groups at a specific position (and not for another) on the scaffold [27]. In that study, none of the ligands with differing binding kinetics exhibited signalling bias for GTPγS binding versus β-arrestin-2 recruitment [27]. Although, in our study, we did not attempt to isolate ligand association and dissociation rates, the signalling kinetics we observed would be consistent with lipophilic ligand CP55,940 having a slower dissociation rate, and therefore, producing more prolonged responses at sub-maximal concentrations than our novel low lipophilicity ligands.

Ligand association rates and the overall time a receptor is occupied by ligand (considering there may be multiple dissociation and re-binding events) can be influenced by ligand interactions with the plasma membrane and by the pathways taken by the ligand to enter and exit the ligand binding pocket. Lipophilic ligands tend to partition into the plasma membrane, which can facilitate the membrane acting as a reservoir retaining a high concentration of ligand near to receptors, and thereby enhance likelihood of initial binding and re-binding [28,29]. This could feasibly manifest as lipophilic ligands producing more prolonged responses in comparison with polar ligands that will tend to diffuse away from the plasma membrane. It is well established that lipophilic CB2 ligands enter the binding site via a membrane-embedded channel/vestibule, and that low affinity interactions along this channel are involved in facilitating orthosteric binding [30,31,32]. It would be interesting to investigate further whether relatively polar ligands, such as those studied here, can enter CB2 via this membrane-embedded channel or must enter directly from the extracellular milieu.

Allosteric modulators can alter the kinetics of signalling responses to orthosteric ligands, e.g., [25,33]. An intriguing possibility is that the novel ligands here studied might produce differential signalling kinetics at CB2 via an allosteric mechanism, i.e., act in an ago-allosteric manner. Competition radioligand binding results indicate that the most energetically favourable binding pose for these ligands overlaps with CP55,940 orthosteric binding. Therefore, if an allosteric mechanism is contributing to the kinetic signalling profiles observed, the ligands might be acting “dualsterically” with an allosteric site in close spatial proximity to the orthosteric site, have transient allosteric interactions, or be acting via dual binding at the orthosteric site and a distant allosteric site on the same or dimerised receptor protomer [34,35,36,37]. Various allosteric sites in CB2 have been predicted, including at least one near the orthosteric site [31,38,39,40,41]. Interestingly, all the test compounds we studied had a considerably higher signalling potency:affinity ratio than CP55,940, which could also indicate involvement of an allosteric mechanism in the transduction of signalling for the test compounds [37].

The temporal and spatial organisation of receptor-proximal signal activation influences downstream integration of signal cascades and subsequent functional outcomes [25,42]. Therefore, it will likely be important to consider the impact of differential spatiotemporal signalling in the ongoing development of CB2 ligands as therapeutics. While prolonged ligand residence time and/or signalling have been associated with improved clinical efficacy in some other GPCRs, this can also increase risk of on-target adverse effects and/or tolerance [43,44,45]. To the authors’ knowledge, the downstream functional impact of differing signalling temporal dynamics and/or partial agonism for CB2 is unknown as yet. We suggest that utilising assays that allow detection of both signalling kinetics and partial agonism may well be important in early characterisation of ligands for potential therapeutic development. We anticipate that recognition of ligands with differential efficacies and temporal signalling patterns, such as those described here, will facilitate direct investigation of the clinical consequences of such properties, and thereby, inform drug development efforts.

## 4. Materials and Methods

### 4.1. Compound Synthesis and Parameters

Details of compound synthesis and compound characterisation are provided in the Supplementary Information.

The logarithm of octanol-water partition coefficient (cLogP) and cLogD_7.4_ were calculated by MarvinSketch (version 22.22, ChemAxon) using the “ChemAxon” method with default electrolyte concentrations (Cl^−^ 0.1 mol/dm^3^, Na^+^ K^+^ 0.1 mol/dm^3^). Higher values indicate higher lipophilicity and, therefore, lower polarity. LogD is similar to LogP, however, the partition is a function of the pH, allowing the ionisation state of compounds at different pH values to be accounted for in lipophilicity measurements. The total polar surface area (TPSA) was estimated using the Polar Surface Area (2D) calculator in MarvinSketch, and is of the unionised species.

### 4.2. Competition Radioligand Binding Assay

Preparation of hCB1- and hCB2-membranes from a HEK pplss-3HA-hCB1 cell line and HEK Flp-in HA-3TCS-hCB2 63R cell line, respectively, followed the protocol described previously [7]. Homologous and heterologous competition radioligand binding assay protocols were equivalent to prior publications [7,46] and were utilised to determine the K_d_ of CP55,940 (Cayman Chemical, Ann Arbor, MI, USA) and K_i_ values of the novel cannabinoids, respectively. A final concentration of 0.75 nM [^3^H]-CP55,940 (Perkin Elmer, Waltham, MA, USA) was used as it met required homologous binding assay assumptions [47]. [^3^H]-CP55,940, hCB1- and hCB2-membranes, and serial dilutions of displacers were separately prepared in binding buffer (50 mM HEPES pH 7.4, 1 mM MgCl_2_, 1 mM CaCl_2_, 2 mg/mL fatty acid-free bovine serum albumin (BSA; ICPbio, Auckland, New Zealand), Milli-Q [MQ] water). Furthermore, 1 µM CP55,940 and vehicle (DMSO) conditions were also used in the heterologous binding assays to assist in defining the maximum displacement window. The concentration of DMSO was kept equivalent across all test conditions. All dilutions were dispensed into a 96-well, polypropylene V-bottom plate (Gene Era Biotech, Hangzhou, China). For each well, the order of dispensing was [^3^H]-CP55,940, displacer, then the hCB1- or hCB2-membrane preparation dilution (10 µg/pt and 2.5 µg/pt, respectively). All conditions were carried out in technical duplicate. The plate was then sealed, tap-mixed, then incubated at 30 °C for 1 h. A total of 50 µL of 0.1% PEI (Sigma-Aldrich, St. Louis, MI, USA) in MQ water was added to each well of a 96-well GF/C harvest plate (Perkin Elmer) 1 h prior to the harvest. When the incubation finished, vacuum (~5 mmHg) was applied to the harvest plate and wells were washed (50 mM HEPES pH 7.4, 500 mM NaCl, 1 mg/mL BSA, MQ water, ice cold). Subsequently, the contents of the V-bottom plate were transferred to corresponding wells in the harvest plate, wash buffer was used to wash the remaining contents of the V-bottom wells before transferring this to the harvest plate, then the harvest plate was washed an additional three times. The plate was dried for ~24 h at 24 °C. To prepare for reading, the bottom of the plate was sealed, each well received 50 µL of Irgasafe Plus scintillation fluid (Perkin Elmer), followed by sealing the top of the plate. Plates were read in a Wallac MicroBeta^®^ TriLux Liquid Scintillation Counter (Perkin Elmer) with a read time of 2 min/well.

GraphPad Prism (v8.0.2; GraphPad Software, San Diego, CA, USA) was used to fit nonlinear regression curves (One site—Fit Ki) to the corrected counts per minutes (CCPM) of each displacer to determine their pK_i_ (negative log K_i_). The K_d_ for the radioligand, [^3^H]-CP55,940, was set to 10.2 nM and 2.7 nM for assays utilising hCB1- and hCB2-membrane preparations, respectively. A 10^−6^ M CP55,940 condition and vehicle-only (DMSO) condition were used to define the bottom and top of the curves, respectively. Mean and SEM of pK_i_ values were calculated from independent experiments. Compounds with concentration-dependent displacement of [^3^H]-CP55,940 to ≥ 75% relative to 1 µM CP55,940 were classed as showing full displacement, while < 75% displacement were classed as partial displacement. Compounds with no displacement or partial displacement of [^3^H]-CP55,940 were considered to only have estimated pK_i_ values at the given receptor. CB2 selectivity was calculated as CB2 K_i_ (or K_d_)/CB1 K_i_ (or K_d_).

### 4.3. cAMP and β-Arrestin-2 Assay Cell Plating and Transfection

HEK-293S cells (HEK-S; generously provided by Professor David Poyner, Aston Research Centre, RRID:CVCL_A784 [48]) were maintained in T75 flasks (Corning-Costar) with Dulbecco’s Modified Eagle’s Medium high glucose with phenol red, L-glutamine and sodium pyruvate (DMEM; Cytiva SH30243), and 8% foetal bovine serum (FBS; NZ-origin, Moregate Biotech) at 37 °C in a humidified atmosphere containing 5% CO_2_.

Poly-D-Lysine (PDL) (Sigma-Aldrich) at 0.05 mg/mL in PBS was incubated in wells of 96-well Solid White Flat Bottom Polystyrene TC-treated Microplate (Corning-Costar) at 37 °C for 1 h, then washed with PBS prior to seeding and transfection. Cells were seeded at 45,000 cells/well in DMEM supplemented with 12% FBS.

Plasmids encoding human CB2 (HA-3TCS-hCB2 63R pcDNA5/FRT; 28.5 ng per well) or no receptor (pcDNA5/FRT/CAT; 23.5 ng per well; Thermo Fisher Scientific, Waltham, MA, USA), and either His-CAMYEL (in pcDNA3L; 24 ng per well; ATCC MBA-277; previously described in [49]), or a mix of Rluc8-hβ-arrestin2-Sp1 (in pcDNA3; 0.5 ng per well; previously described in [19]) and Mem-linker-Citrine-SH3 (in pcDNA3; 20 ng per well; previously described in [19,50]) were diluted in DMEM. Diluted DNA was then co-incubated with polyethyleneimine max (PEI max; Polysciences, Warrington, PA, USA) (Total DNA:PEI ratio of 1:7.5 or 1:6.7 for cAMP or β-arrestin-2 experiments, respectively) in DMEM for 1 h at room temperature (~19–21 °C). These transfection mixes were then added to plated cells, resulting in a final concentration of 8% FBS. Plates were incubated for ~48 h before assaying.

### 4.4. cAMP and β-Arrestin-2 Assay Procedure and Analysis

The protocol for measuring levels of cellular cAMP via real-time BRET biosensor (CAMYEL) is adapted from previous publications [7,33]. In all cAMP experiments, forskolin (Tocris Bioscience, Bristol, UK) was used to stimulate the activation of adenylyl cyclase to raise intracellular cAMP levels. Vehicle (DMSO) was maintained at an equivalent concentration across all conditions. Assays were carried out in technical duplicate. Then, ~48 h post-transfection, plating media was aspirated, and wells were washed with H+B, consisting of HBSS (Life Technologies) and 1 mg/mL BSA (ICPbio). H+B was then added to the wells to initiate a 30 min serum starve at 37 °C. Post-serum starve, coelenterazine h (final concentration 5 μM, NanoLight Technologies, Norman, OK, USA, #301) was added to the wells, and a 5 min pre-reading was conducted using the LUMIstar*^®^* Omega luminometer (Rluc and YFP emissions detected at 460 nm and 535 nm, respectively; BMG Labtech, Ortenberg, Germany). Next, forskolin (final concentration 5 μM) was added to all wells (except vehicle-only wells which received DMSO), and plate reading resumed for 5 min. Lastly, CB2 compounds of interest with forskolin, vehicle with forskolin, or vehicle without forskolin, were added to their respective wells, and a final 20 min read was conducted. All compounds and reagents were diluted in H+B prior to addition to the plate. For β-arrestin-2 recruitment assays, an equivalent procedure was followed except no forskolin was included in the assay stimulations.

BRET ratios were calculated by dividing YFP emissions by Rluc emissions, then for the cAMP assay, normalised to the mean of baseline ratio reads (where only coelenterazine h was present) to eliminate any variability present prior to drug addiction. An increase in BRET ratio indicated a decrease in cAMP levels or increase in β-arrestin-2 recruitment. Lowess smoothing (GraphPad Prism) was applied to individual technical replicates within an assay. Concentration response curves for the mean of technical replicates (CRC; “log(agonist) vs. response (three parameters)” nonlinear regression curve model, Hill slope constrained to 1) were drawn for the mean ratios over 20 min stimulation or individual time-points. For the cAMP assay, data were normalised to matched vehicle (0%) and ‘top’ of the CRC (near to forskolin-only) (100%), or for the β-arrestin-2 recruitment assay, normalised to the ‘bottom’ of the CRC (near vehicle) (100%), allowing compilation of data from independent experiments. CRCs were re-drawn for independent experiment normalised data, from which E_max_ and pEC_50_ parameters were derived and subsequently parameter means and SEM calculated.

### 4.5. Bias Analysis

Bias analysis was carried out as previously described with minor modifications [7]. In brief, concentration response data from independent experiments at either individual timepoints or for the mean response over 20 min were normalised such that the largest response in the dataset was equal to 100% and the vehicle plus forskolin (cAMP) or vehicle (arrestin) condition was equal to 0%. Normalised data from independent experiments were then fitted with the Black and Leff operational model for bias with equations in GraphPad Prism, as described previously [7,17,18]; constraints were system E_max_ 100%, basal response 0%, ‘n’ transducer function slope 1. For each timepoint or mean 20 min response, ∆LogR was calculated as the difference between LogR for CP55,940 and LogR for the other compounds of interest in the same signalling assay and time-point. The differences between cAMP and arrestin mean ∆LogRs were then calculated to find ∆∆LogR.

### 4.6. Statistical Analysis

GraphPad Prism was used to conduct statistical comparison of binding and signalling mean 20 min parameters. Ordinary one-way ANOVA were conducted, followed by a Holm-Šídák Multiple Comparisons test when comparison to control was of interest (binding), or Tukey’s Multiple Comparisons test to compare all means (signalling). SigmaPlot (v14, Systat Software, San Jose, CA, USA) was used to conduct statistical tests on temporally resolved signalling data. For comparison of parameters over time, one-way ANOVA with repeated measures tests were performed (reflecting that in each independent experiment multiple measurements over time were taken from the same samples), followed by Holm-Šídák Multiple Comparisons test for comparison to the indicated timepoint. When comparison over time between drugs was of interest, a two-way ANOVA was conducted, followed by Holm-Šídák Multiple Comparisons test. Bias data were analysed as for signalling assays, except ordinary one-way ANOVA (without repeated measures) was used for comparison of ∆∆LogR over time. All datasets were tested for normality and equal variance (utilising default tests for each software package); if one or both tests did not pass, data were log transformed, which resulted in the dataset then passing the assumptions for parametric testing.

## Figures and Tables

**Table 1:** Physiochemical properties, human CB2 and CB1 binding affinities, and CB2 selectivity (relative to CB1) for studied compounds. Affinity is provided as pK_d_ for CP 55,940, or pK_i_ for all other compounds. Some parameters are approximate (~, <, >) due to there being incomplete displacement of the radioligand at the maximum compound concentration tested. ^a^ first reported in [6].

Compound	Chemical Structure	Molecular Weight (Da)	TPSA (Å^2^)	cLogP (cLogD_7.4_)	CB2 pK_d_/pK_i_ (±SEM)	CB1 pK_d_/pK_i_ (±SEM)	CB2/CB1 Selectivity
UOSS77/S-777469 ^a^	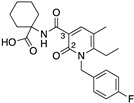	414.5	86.7	3.13 (−0.25)	6.73 (0.04)	~4.80 (0.08)	~80
UOSS75	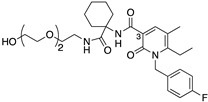	545.7	117.2	1.44 (1.44)	6.83 (0.13)	~4.82 (0.21)	~100
UOSS76	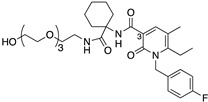	589.7	126.4	1.27 (1.27)	6.09 (0.18)	~4.53 (0.16)	~40
UOMM18	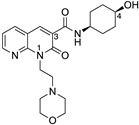	514.5	95.0	−0.19 (−0.20)	6.14 (0.09)	<4	>126
CP55,940	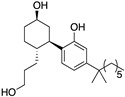	376.6	60.7	5.57 (5.57)	8.58 (0.09)	7.99 (0.12)	4

## Data Availability

The data supporting this study’s findings are available from the corresponding author upon reasonable request.

## Supplementary Materials

The supporting information can be downloaded at: https://www.mdpi.com/article/10.3390/ijms24076406/s1.
