# Supplementary material for: Novel Cannabinoid Receptor 2 (CB2) Low Lipophilicity Agonists Produce Distinct cAMP and Arrestin Signalling Kinetics without Bias
Source: Int J Mol Sci. 2023 Mar 29;24(7):6406. doi: 10.3390/ijms24076406 (PMC10094510; doi:10.3390/ijms24076406)
Supplement: Supplementary file 1 [file ijms-24-06406-s001.zip › ijms-2248873-supplementary.pdf]

Novel Cannabinoid Receptor 2 (CB2) Low Lipophilicity Agonists Produce Distinct cAMP and Arrestin Signalling Kinetics Without Bias

Supplementary Information

Supplementary Figure S1 | cAMP assay concentration response curves at selected time-points, and response over time at 2.5-minute EC_{75} concentration and E_{max} 2

Supplementary Figure S2 | β-arrestin-2 plasma membrane translocation concentration response curves at selected time-points, and response over time at 1.5-minute EC_{75} concentration and E_{max} 4

Supplementary Methods: Compound Synthesis 6

UOSS77 6

UOSS75 7

UOSS76 8

UOMM18 9

Supplementary Information References........ 11

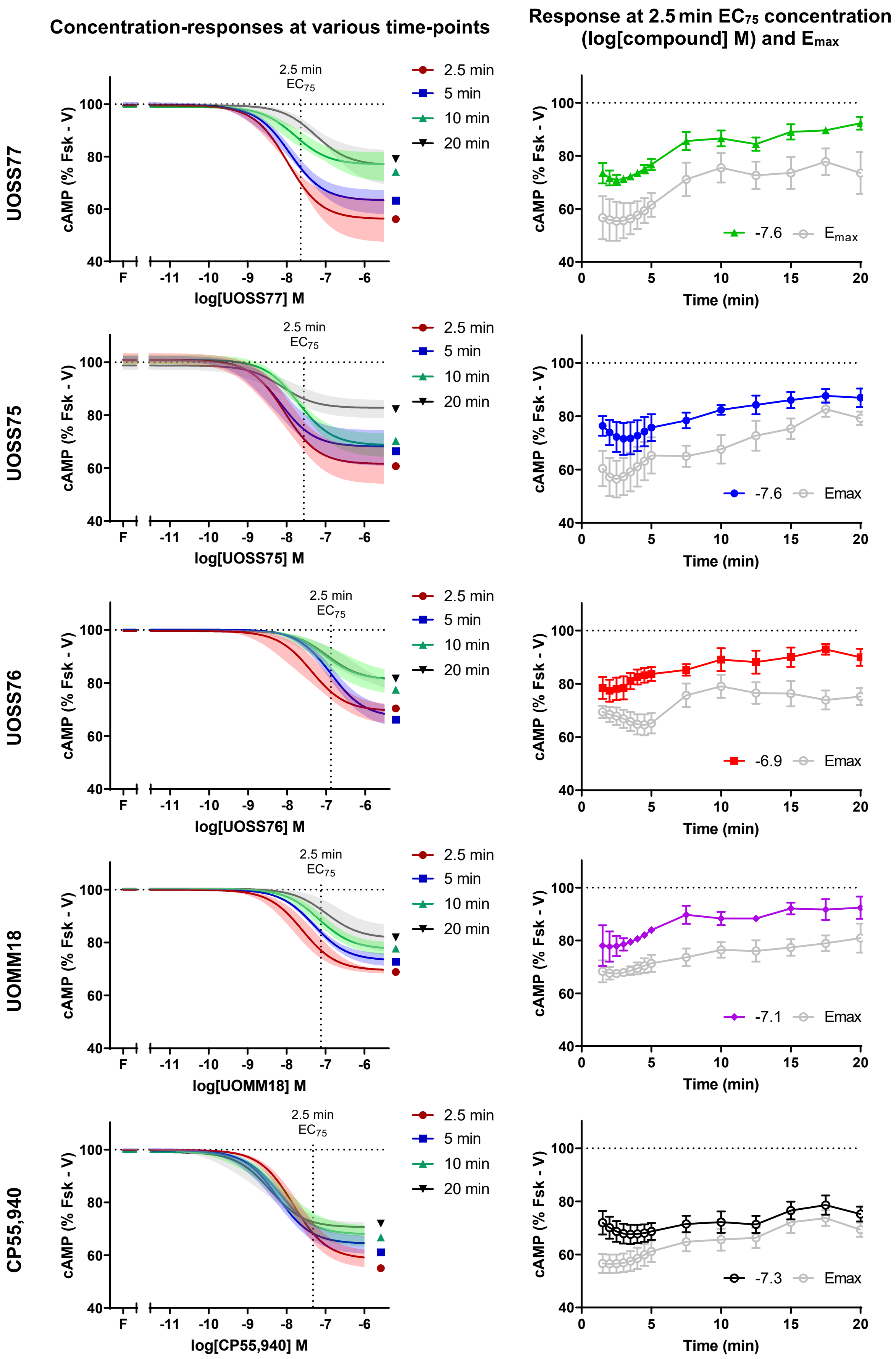

Supplementary Figure S1 | cAMP assay concentration response curves at selected time-points (left), and response over time at 2.5-minute EC_{75} concentration and E_{max} (right). Caption continued next page.

Supplementary Figure S1 continued | Cells were concurrently stimulated with compounds of interest (as indicated) and 5 µM forskolin for differing time periods. Data normalised to vehicle-treated without forskolin (V; 0%) and vehicle-treated with forskolin (F; 100%) as in Figures 2 and 3, such that smaller cAMP measurements indicate greater efficacy (inhibition of cAMP synthesis). Data are mean ± SEM from 3-4 independent experiments. Left-hand graphs are mean and SEM (shading) for sigmoidal curves fitted to independent experiments; vertical dotted lines indicate the 2.5-minute EC_{75} concentrations (based on calculation from mean EC_{50}, Figure 3). Responses for the 2.5-minute EC_{75} concentration derived from the fitted sigmoidal curves (as in left-hand panels) and E_{max} (from Figure 3) are plotted over time on the right-hand panel.

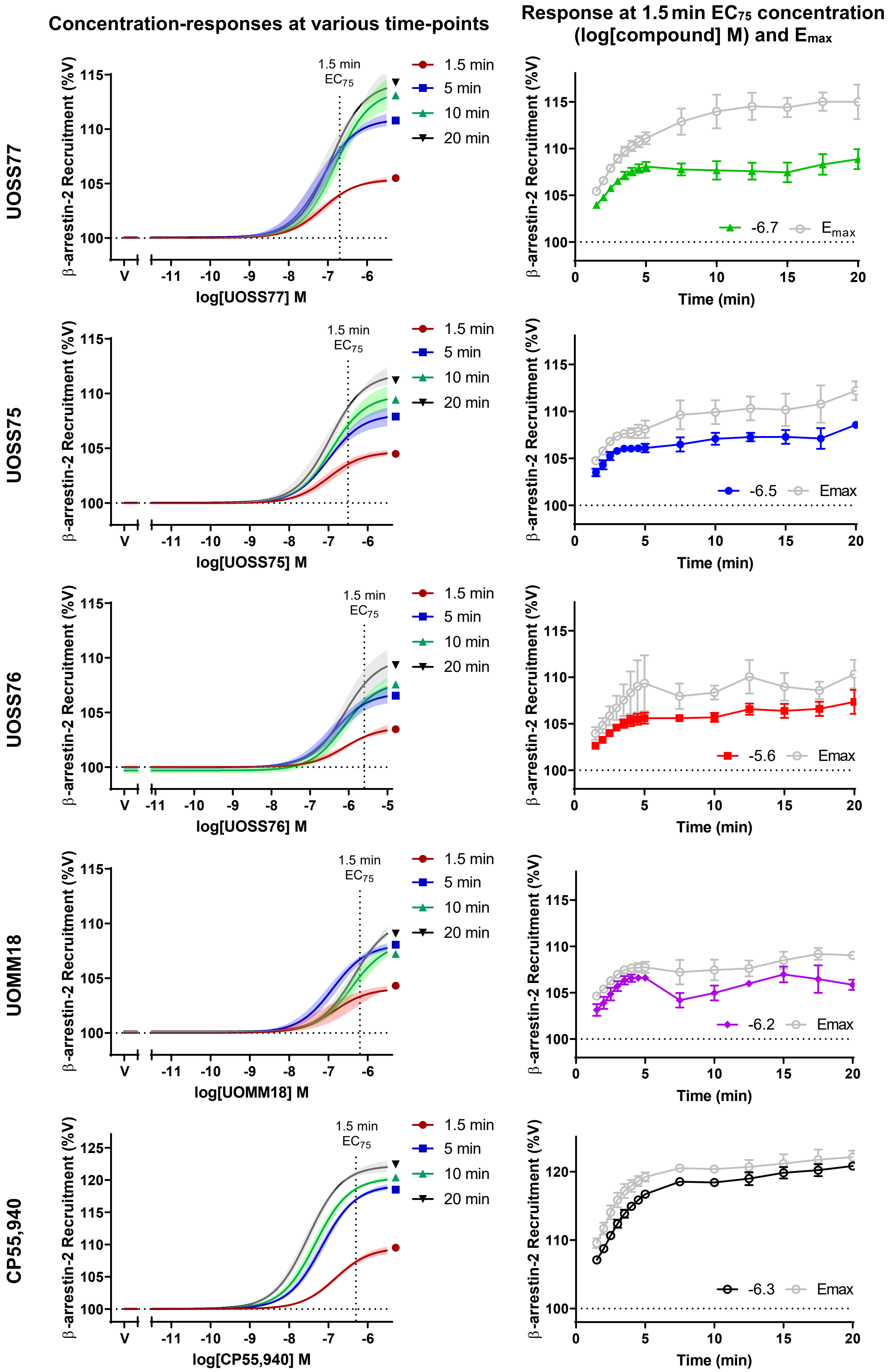

Supplementary Figure S2 | β-arrestin-2 plasma membrane translocation concentration response curves at selected time-points (left), and response over time at 1.5-minute EC_{75} concentration and E_{max} (right). Caption continued next page.

Supplementary Figure S2 continued | Cells were stimulated with compounds of interest for differing time periods as indicated. Data normalised to vehicle-treated (V; 100%) as in Figures 4 and 5, such that larger E_{max} values indicate greater efficacy. Data are mean ± SEM from 3 independent experiments. Left-hand graphs are mean and SEM (shading) for sigmoidal curves fitted to independent experiments; vertical dotted lines indicate the 1.5-minute EC_{75} concentrations (based on calculation from mean EC_{50}, Figure 5). Responses for the 1.5-minute EC_{75} concentration derived from the fitted sigmoidal curves (as in left-hand panels) and E_{max} (from Figure 5) are plotted over time on the right-hand panel. Note that CP55,940 has a different Y-axis range from the test compounds.

Supplementary Methods: Compound Synthesis

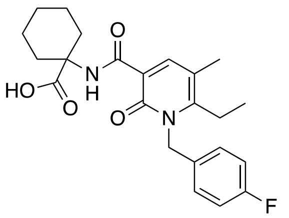

1-(6-ethyl-1-(4-fluorobenzyl)-5-methyl-2-oxo-1,2-dihydropyridine-3-carboxamido)cyclohexane-1-carboxylic acid **UOSS77**

Compound **UOSS77** was prepared as previously reported in [1] (compound **S-777469**) and patent [2] (compound **VIII-a**) with one modification. Exactly as previously reported, commercially available starting materials **1**, **2** and **3** gave acid **4** in 3 steps. At this stage, instead of an acid chloride-mediated transformation of **4** to **5** as previously reported [1], a tetramethylfluoroformamidinium hexafluorophosphate (TFFH)-mediated coupling (detailed below) was undertaken to transform **4** to **5**. Conversion of **5** to **UOSS77** was again exactly as previously reported [1,2].

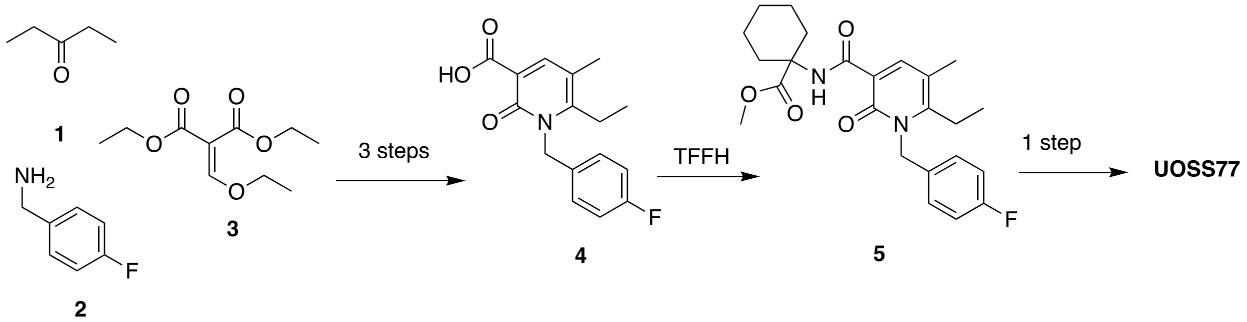

Methyl 1-(6-ethyl-1-(4-fluorobenzyl)-5-methyl-2-oxo-1,2-dihydropyridine-3-carboxamido)cyclohexane-1-carboxylate **5**.

To a mixture of **4** (0.70 g, 2.42 mmol) and TFFH (0.64 g, 2.42 mmol) in DMF (20 mL) at 0°C was added DIPEA (0.41 g, 7.26 mmol) and the mixture was stirred for 1h. Then, methyl 1-aminocyclohexane-1-carboxylate (0.38 g, 2.42 mmol) was added and the reaction was allowed to warm naturally to RT and was stirred for 12 h. The DMF was reduced by evaporation under reduced pressure. The residue was partitioned between EtOAc (50 mL) and H_2O (50 mL). The organic layer was then washed twice with water (50 mL), dried over $MgSO_4.H_2O$ and concentrated under reduced pressure. This residue was purified by silica column chromatography (eluent 1:1 ethyl acetate: hexane) to give **5** (0.93 g, 2.17 mmol, 90%) as a pale yellow solid.

NMR data for **S-777469** was not given in [1]. However, ^{1}H NMR spectral data of **UOSS77** is consistent with previous patent report (compound VIII-a in [2]).

UOSS77 ^{1}H NMR (400 MHz, DMSO-d_6) δ 1.04 (t, 3H, J = 7.5 Hz, CH_3), 1.13 – 1.46 (m, 4H, 2 × CH_2), 1.47 – 1.62 (m, 2H, CH_2), 1.63 – 1.77 (m, 2H, CH_2), 1.93 – 2.09 (m, 2H, CH_2), 2.18 (s, 3H, CH_3), 2.66 (q, 2H, J = 7.5 Hz, CH_2), 5.44 (s, 2H, CH_2), 7.07 – 7.24 (m, 4H, ArH), 8.19 (s, 1H, ArH), 10.13 (s, 1H, NH), 12.30 (br s, 1H, CO_2H). ^{13}C NMR (101 MHz, DMSO-d_6) δ 11.85, 16.78, 21.20, 23.50, 24.85, 31.90, 46.53, 57.64, 113.82, 115.67, 116.95, 128.10, 132.98, 145.49, 153.94, 160.05, 161.78, 162.08, 175.30.

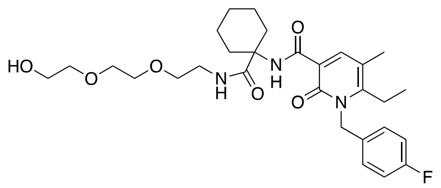

6-Ethyl-1-(4-fluorobenzyl)-N-(1-((2-(2-(2-hydroxyethoxy)ethoxy)ethyl)carbamoyl)cyclohexyl)-5-methyl-2-oxo-1,2-dihydropyridine-3-carboxamide **UOSS75**

A solution of **UOSS77** (26 mg, 0.07 mmol), TFFH (18 mg, 0.07 mmol), Et_3N (0.03 mL, 0.14 mmol) in DMF (2.0 mL) under N_2 atmosphere was stirred for 10 min. A solution of *N*-hydroxysuccinimide (9 mg, 0.07 mmol) in DMF (1.0 mL) was then added and the reaction stirred for 30 min. A solution of 2-[2-(2-aminoethoxy)ethoxy]ethan-1-ol (10 mg, 0.07 mmol in DMF [1.0 mL]) was then added and the reaction stirred for 12 h. The reaction solvent was removed under reduced pressure. The residue was partitioned between EtOAc (10 mL) and H_2O (10 mL). The organic layer was then washed with water (10 mL), brine solution (10 mL), dried over $MgSO_4.H_2O$ and concentrated under reduced pressure. The residue obtained was washed with diethyl ether and hexane and concentrated under reduced pressure to provide **UOSS75** (18mg, 0.03 mmol, 49%) as a clear liquid. ^{1}H NMR (400 MHz, $CDCl_3$) δ 1.15 (t, 3H, J = 7.5 Hz, CH_3), 1.20 – 1.34 (m, 2H, CH_2), 1.37 – 1.54 (m, 2H, CH_2), 1.56 – 1.70 (m, 2H, CH_2), 1.78 – 1.94 (m, 2H, CH_2), 2.20 (s, 3H, CH_3), 2.22 – 2.36 (m, 2H, CH_2), 2.65 (q, 2H, J = 7.5 Hz, CH_2), 3.40 – 3.49 (m, 2H, CH_2), 3.51 – 3.63 (m, 8H, 4 × CH_2), 3.64 – 3.73 (m, 2H, CH_2), 5.44 (s, 2H, CH_2), 6.96 – 7.12 (m, 4H, ArH), 7.34 (t, 1H, J = 5.6 Hz, NH), 8.37 (s, 1H, ArH), 10.20 (s, 1H, NH). ^{13}C NMR (101 MHz, $CDCl_3$) δ 12.27, 17.40, 21.72, 24.01, 25.47, 32.30, 39.46, 47.09, 60.72, 61.91, 70.16, 70.47, 70.52, 72.75, 114.44, 115.98, 118.02, 127.87, 131.84, 146.65, 153.42, 161.07, 162.63, 164.23, 175.22. HRMS calculated for $C_{29}H_{40}FN_3NaO_6$ $[M + Na]^+$, 568.2793; found, 568.2797.

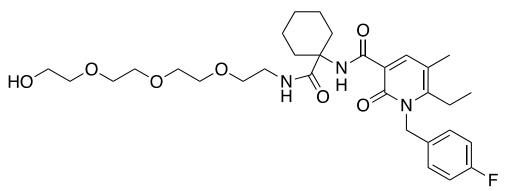

6-Ethyl-1-(4-fluorobenzyl)-N-(1-((2-(2-(2-(2-hydroxyethoxy)ethoxy)ethoxy)ethyl)carbamoyl)cyclohexyl)-5-methyl-2-oxo-1,2-dihydropyridine-3-carboxamide **UOSS76**

Compound **UOSS76** was prepared following the same procedure described for **UOSS75**, using **UOSS77** (0.35 mg, 0.08 mmol) and 2-{2- [2- (2- aminoethoxy)ethoxy]ethoxy}ethan- 1- ol (17 mg, 0.08 mmol). Compound **UOSS76** (44 mg, 0.07 mmol, 84%) was obtained as a clear oil. ^{1}H NMR (400 MHz, $CDCl_3$) δ 1.15 (t, 3H, *J* = 7.6 Hz, CH_3), 1.20 – 1.35 (m, 2H, CH_2), 1.38 – 1.55 (m, 2H, CH_2), 1.56 – 1.69 (m, 2H, CH_2), 1.80 – 1.94 (m, 3H, CH_2, NH), 2.19 (s, 3H, CH_3), 2.21 – 2.31 (m, 2H, CH_2), 2.65 (q, 2H, *J* = 7.5 Hz, CH_2), 3.45 (q, 2H, *J* = 5.3 Hz, CH_2), 3.51 – 3.71 (m, 14H, 7 × CH_2), 5.43 (s, 2H, CH_2), 6.99 – 7.12 (m, 4H, ArH), 7.29 (t, 1H, *J* = 5.6 Hz, NH), 8.37 (s, 1H. ArH), 10.20 (s, 1H, N**H**). ^{13}C NMR (101 MHz, $CDCl_3$) δ 12.30, 17.39, 21.77, 24.01, 25.00, 32.29, 39.0, 47.07, 60.54, 61.72, 70.29, 70.33, 70.35, 70.62, 72.66, 114.37, 115.99, 118.13, 127.95, 131.92, 146.63, 153.32, 161.07, 162.62, 163.98, 175.22. HRMS calculated for $C_{31}H_{44}FN_3NaO_7$ [M + Na]$^+$, 612.3055; found, 612.3025.

N-((1s,4s)-4-Hydroxycyclohexyl)-1-(2-morpholinoethyl)-2-oxo-1,2-dihydro-1,8-naphthyridine-3-carboxamide **UOMM18**

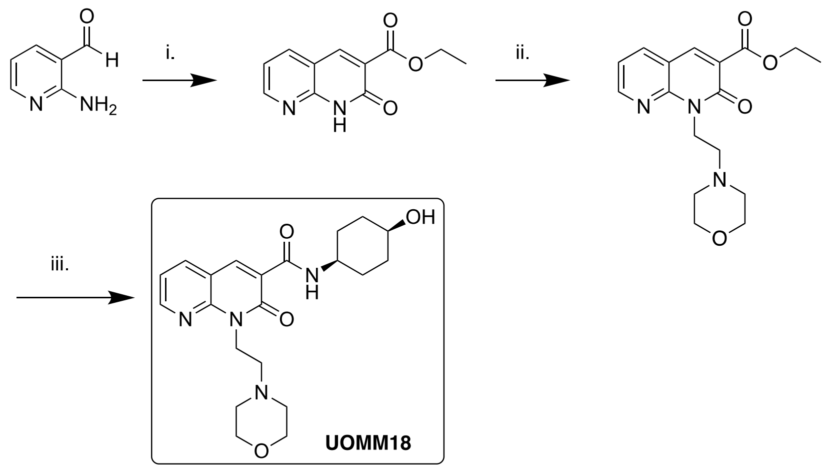

Ethyl 2-oxo-1,2-dihydro-1,8-naphthyridine-3-carboxylate

2-Aminopyridin-3-carboxaldehyde (1.0 g, 8.19 mmol) and diethyl malonate (1.967 g, 12.28 mmol) were dissolved in ethanol (20 mL) and piperidine (0.24 mL) was added. After stirring for 20 h at 50°C, followed by cooling to RT, the solid that formed was filtered, washed with diethyl ether, and recrystallised from ethyl acetate to give ethyl 2-oxo-1,2-dihydro-1,8-naphthyridine-3-carboxylate (1.35 g, 6.2mmol, 75%) as dark yellow needles. ^{1}H NMR (400 MHz, DMSO-d_6) δ 1.30 (t, 3H, *J* = 7.1 Hz, CH_3), 4.28 (q, 2H, *J* = 7.1 Hz, CH_2), 7.29 (dd, 1H *J* = 7.8, 4.8 Hz, ArH), 8.27 (dd, 1H *J* = 7.8, 1.9 Hz, ArH), 8.49 (s, 1H, ArH), 8.60 (dd, 1H *J* = 4.8, 1.9 Hz, ArH), 12.43 (s, 1H, NH). ^{13}C NMR (101 MHz, DMSO) δ 14.11, 60.88, 112.94, 118.79, 124.64, 138.12, 142.46, 150.39, 152.70, 159.28, 163.99. HRMS-ESI calculated for $C_{11}H_{10}N_2NaO_3$ $[M+Na]^+$ 241.0584; found 241.0591.

Ethyl 1-(2-morpholinoethyl)-2-oxo-1,2-dihydro-1,8-naphthyridine-3-carboxylate

Ethyl 2-oxo-1,2-dihydro-1,8-naphthyridine-3-carboxylate (1.3 g, 5.96 mmol) was dissolved in DMF (20 mL) and cesium carbonate (5.44 g, 16.7 mmol) was added. After stirring for 1 h at RT, 4-(2-chloroethyl)-morpholine.HCl (2.2 g, 11.9 mmol) was added and the mixture was stirred at 50°C for 12 h. The mixture was cooled to RT and the solvent evaporated under reduced pressure. To the crude residue, a solution of aqueous saturated sodium bicarbonate (50 mL) was added and extracted with CH_2Cl_2 (3 x 50 mL) and dried over $MgSO_4$, and the solvent evaporated. This yielded a brown oil that was recrystalised from ethyl acetate to give ethyl 1-(2-morpholinoethyl)-2-oxo-1,2-dihydro-1,8-naphthyridine-3-carboxylate (1.7 g, 5.2 mmol, 87%) as red crystals. ^{1}H NMR (400 MHz, $CDCl_3$) δ 1.42 (t, 3H, J = 7.2 Hz, CH_3), 2.50 – 2.88(m, 6H, CH_2), 3.59 – 3.80 (m, 4H, CH_2), 4.43 (q, 2H, J = 7.2 Hz, CH_2), 4.67 – 4.81 (m, 2H, CH_2), 7.23 (dd, 1H, J = 7.7, 4.7 Hz, ArH), 7.97 (dd, 1H, J = 7.7, 1.9 Hz, ArH), 8.36 (s, 1H, ArH), 8.67 (dd, 1H, J = 4.7, 1.9 Hz, ArH). ^{13}C NMR (101 MHz, $CDCl_3$) δ 14.62, 38.93, 54.18, 56.07, 62.10, 67.33, 114.36, 118.80, 124.59, 138.30, 142.04, 150.90, 152.47, 159.62, 164.83. HRMS-ESI calculated for $C_{17}H_{22}N_3O_4$ $[M+H]^+$ 332.1605; 332.

cis-N-(4-Hydroxycyclohexyl)-1-(2-morpholinoethyl)-2-oxo-1,2-dihydro-1,8-naphthyridine-3-carboxamide **UOMM18**

A mixture of ethyl 1-(2-morpholinoethyl)-2-oxo-1,2-dihydro-1,8-naphthyridine-3-carboxylate (0.35 g, 1.05 mmol) and cis-4-aminocyclohexanol (0.60 g, 5.25 mmol) were heated without solvent to 80°C in a pressure tube for 52 hours. After cooling, water was added and the mixture extracted with CH_2Cl_2, dried over $MgSO_4$ and evaporated under reduced pressure to give a crude residue that was purified by silica column chromatography (CH_2Cl_2/MeOH 92:8) to afford **UOMM18** (247 mg, 0.62 mmol, 59%) as pale yellow crystals. ^{1}H NMR (400 MHz, $CDCl_3$) 1.66 – 1.89 (m, 8H, 4 × CH_2), 2.63 (m, 4H, 2 × CH_2), 2.74 (t, 2H, J = 7.1 Hz, CH_2), 3.68 (t, 4H, J = 4.6 Hz, 2 × CH_2), 3.90 (m, 1H, CH), 4.11 (m, 1H, CH), 4.76 (t, 2H, J = 7.1 Hz, CH_2), 7.27 (m, 1H ArH), 8.06 (dd, 1H, J = 7.8, 1.9 Hz, ArH), 8.68 (dd, 1H, J = 4.7, 1.9 Hz, ArH), 8.85 (s, 1H, ArH), 9.90 (d, 1H, J = 8.4 Hz, NH). ^{13}C NMR (101 MHz, $CDCl_3$) δ 27.50, 31.35, 38.73, 46.43, 53.83, 55.79, 66.89, 67.08, 114.86, 119.06, 123.01, 138.42, 141.93, 149.82, 151.97, 161.98, 162.56. HRMS-ESI calculated for $C_{21}H_{29}N_4O_4$ $[M+H]^+$ 401.2183; found 401.2154.

Supplementary Information References

1. Odan, M.; Ishizuka, N.; Hiramatsu, Y.; Inagaki, M.; Hashizume, H.; Fujii, Y.; Mitsumori, S.; Morioka, Y.; Soga, M.; Deguchi, M.; et al. Discovery of S-777469: An Orally Available CB2 Agonist as an Antipruritic Agent. Bioorganic Med. Chem. Lett. 2012, 22, 2803–2806. https://doi.org/10.1016/j.bmcl.2012.02.072.

2. Shionogi & Co. Process for Producing 1-Substituted-2-Pyridone-3-Carboxylic Acid Derivative (Patent WO2008084671) 2008. Available online: https://web.archive.org/web/20230122064416/https://patentscope.wipo.int/search/en/detail.jsf?docId=WO2008084671&_cid=P21-LA1KFF-65044-1 (accessed on 22 January 2023).
